# Single Applications of Commercial Mammal Deterrents Fail to Prevent Chewing Damage to Passive Acoustic Sensors

**DOI:** 10.3390/s26154704

**Published:** 2026-07-24

**Authors:** Brooke D. Goodman, Lauren M. Chronister, Tessa A. Rhinehart, R. Patrick Lyon, Justin Kitzes

**Affiliations:** Department of Biological Sciences, University of Pittsburgh, Pittsburgh, PA 15260, USA; lauren.chronister@pitt.edu (L.M.C.); tessa.rhinehart@pitt.edu (T.A.R.); rpl21@pitt.edu (R.P.L.); justin.kitzes@pitt.edu (J.K.)

**Keywords:** bioacoustics, remote sensing, autonomous recording unit, AudioMoth, acoustic monitoring

## Abstract

Large sensor arrays are an increasingly popular sampling method among ecologists. To last in the field, sensor housing needs to be resistant to damage from both weather and animals. The popular AudioMoth acoustic recorder does not have integral weather-resistant housing and is deployed by users in a wide variety of protective cases. One inexpensive way to protect AudioMoths is to deploy them in plastic bags, which offer moderate weather resistance but are susceptible to chewing damage from small mammals. In this study, we test the effectiveness of commercially available mammal deterrents in preventing such chewing damage. We deployed 115 treatment-control pairs across two grids in temperate forests in Pennsylvania. Bag treatments consisted of Liquid Fence, Bonide, and a cayenne and Vaseline mixture. For all deterrents, there was no statistically significant difference in the proportion or severity of mammal chewing damage between treatments and controls. Counter to expectations, for all three treatments, more of the bags treated with a deterrent were damaged by mammal chewing than the paired control bags. Our results strongly suggest that single applications of these three deterrents have no useful effect on preventing mammal chewing damage to sensor housing in the field.

## 1. Introduction

Remote sensors allow for data collection at spatiotemporal scales previously impossible in ecology [1]. The deployment of large sensor arrays, an increasingly popular sampling method among ecologists, necessitates low-cost, lightweight, and small sensors that can be purchased, packed, and deployed in large quantities. For acoustic recorders, these specifications are met by units such as the AudioMoth, a 5 g, 58 × 48 × 15 mm recording unit, capable of capturing months of audio data in a single deployment. Deploying any sensor for long periods requires that its housing is both resilient enough to endure adverse weather and curious animals, and acoustically permeable so sound can reach the internal microphone.

**Figure 1:**
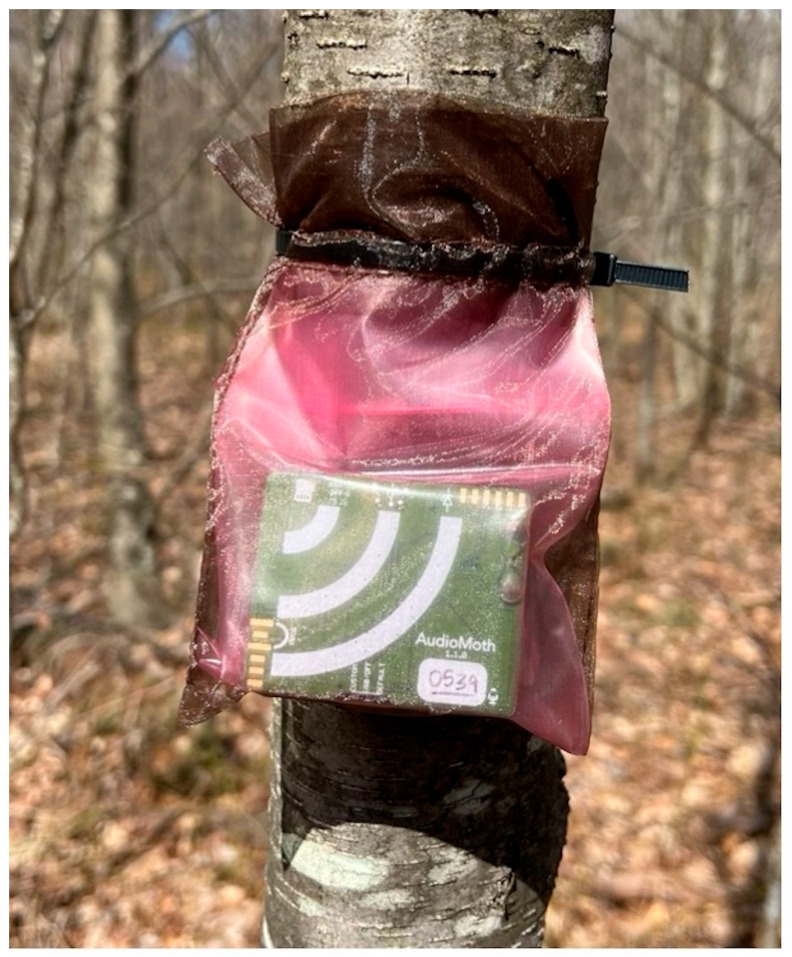
An AudioMoth deployed in the reclosable, chiffon bag apparatus, zip-tied to a tree.

The AudioMoth is manufactured without an integral housing, placing the onus on the user to choose appropriate materials to protect these devices in the field. OpenAcoustics, the supplier of the AudioMoth, offers a waterproof case that can be purchased separately from the recorder itself, but this case has been found to alter the frequency response and directionality of the microphone, an issue for deployments aiming to capture sound omnidirectionally [2]. An alternative option is to house AudioMoths in plastic bags, which have been found to be acoustically transparent and provide moderate protection from weather damage [2]. Our group, for example, frequently deploys AudioMoth recorders in 4 × 6 in, 4 mm thick anti-static reclosable bags, which are then placed in chiffon bags used to secure the apparatus to a tree or post via zip ties (Figure 1). This lightweight, flexible protective bag allows for the deployment of dozens of recorders in a single trip, with little added weight to field packs and minimal sound attenuation at the internal microphone [3].

This housing approach has proved generally successful for large-scale deployments in temperate forests [4,5]. However, it leaves recorders vulnerable to damage from small mammals chewing through the bag, which can lead to water ingress and damage the recorder itself. Chewing damage often presents as small, repeated bite marks along the bottom of the chiffon bag, penetrating the reclosable bag, and allowing moisture into the housing. One deployment of 196 AudioMoths in the housing described above from April to June 2024 in a temperate Pennsylvania forest resulted in 12% of the recorders being damaged due to moisture ingress caused by mammal chewing.

Commercially available and commonly used mammal deterrents abound, but their effectiveness in protecting electronic sensors in the field has received little study. In multiple studies using telecommunication cables and decorative landscaping features, capsaicin mixed into a wax-like substance and applied to target surfaces effectively reduced rodent gnawing [6,7,8]. Capsaicin coatings have also been found to reduce mammals’ seed consumption in the contexts of restoration seeding and bird feeding [9,10,11,12,13]. Other studies have had mixed success in protecting equipment in the field using liquid mammal deterrents containing ingredients like denatonium saccharide, egg solids, and garlic oil to reduce mammal chewing behavior [14,15].

Here, we aim to test the effectiveness of single applications of cayenne pepper (capsaicin) mixed with Vaseline, Liquid Fence, and Bonide spray in reducing mammal chewing behavior over a month-long field deployment. We hypothesized that AudioMoths housed in reclosable and chiffon bags treated with any deterrent would have less damage from small mammals than untreated controls. To test this hypothesis, we conducted 115 paired deployments of treated and untreated AudioMoths in a temperate forest in Conneaut Lake, Pennsylvania. Our specific objectives were to (1) compare the damage rates of treated and untreated AudioMoths; (2) compare damage rates among different treatment types; and (3) compare damage intensity between different treatments. Here we report the findings of our field trial and offer suggestions for further research on mammal deterrents in field settings.

## 2. Materials and Methods

### 2.1. Materials

We deployed 115 treatment-control pairs (230 AudioMoths, all deemed unusable due to damage from previous deployments) to simulate a large recording array for this study. Each AudioMoth was placed in the housing described in the introduction, which involved installing three dead AA batteries, placing the AudioMoth in a new reclosable bag with a silica gel pack, and then placing that reclosable bag within a chiffon bag. Reclosable bags were made of 100% linear low-density polyethylene. AudioMoths were zip-tied to trees via a loop in the chiffon bag. The AudioMoths were not programmed to record and were not turned on.

### 2.2. Experimental Design

**Figure 2:**
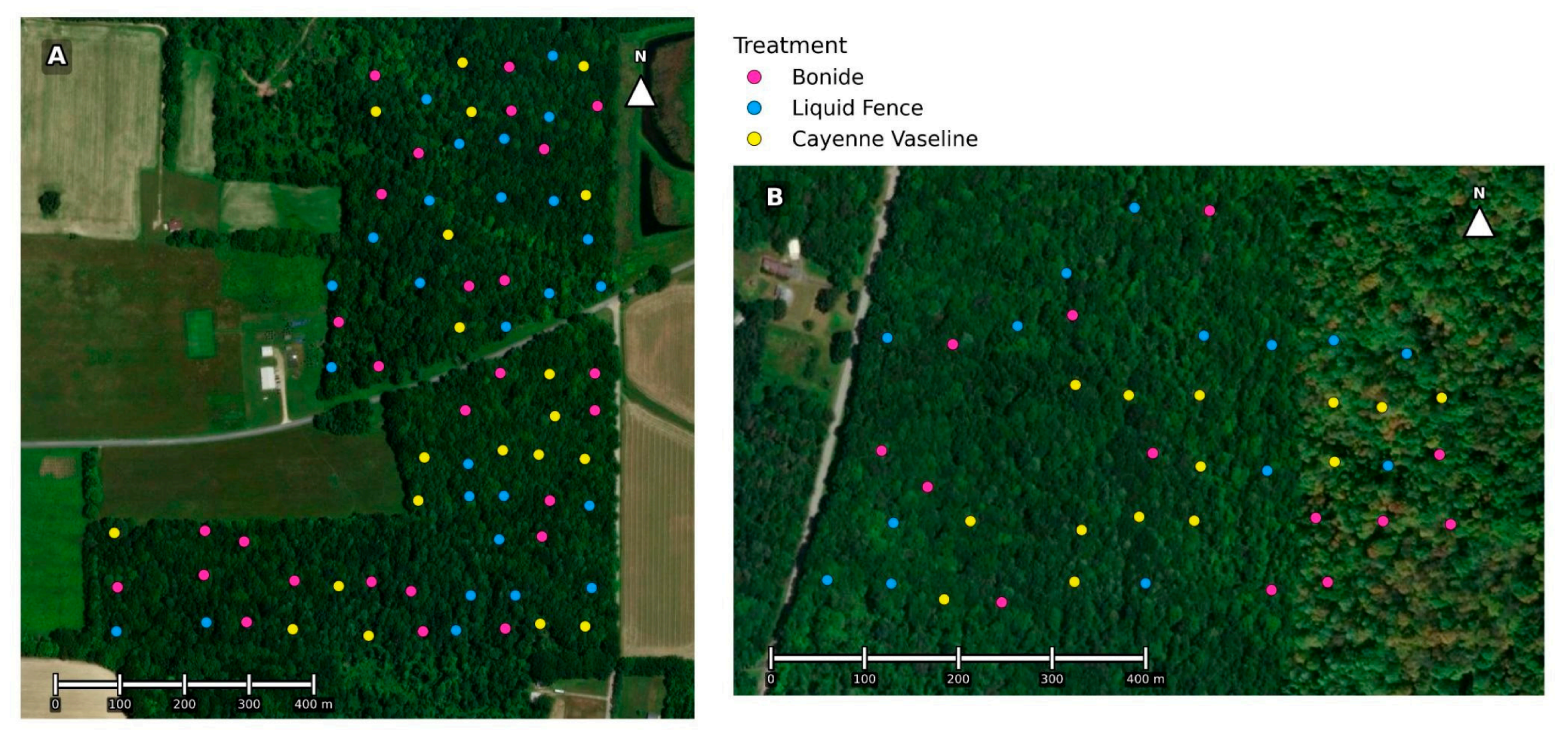
Two recorder arrays deployed in Conneaut Lake, Pennsylvania. (**A**) Array A consists of 74 points. (**B**) Array B consists of 41 points. Each point represents a tree on which two AudioMoths, a treatment and a control, were co-deployed. The arrays were separated by 12 km.

AudioMoths were deployed at two arrays in a temperate Pennsylvania forest from 17 to 23 September and retrieved from 17 to 24 October 2024 (Figure 2). One array was located in the Janette Rose Tryon Preserve and the other at the Donald S. Wood Laboratory, both of which are part of the Pymatuning Laboratory of Ecology, owned by the University of Pittsburgh. Signs of many mammals which we suspect cause damage to AudioMoths were evident on these properties and included direct observations of species such as Eastern gray squirrel (*Sciurus carolinensis*), Fox squirrel (*Sciurus niger*), and Eastern chipmunk (*Tamias striatus*). The den and scat of a fox species (either Red fox *Vulpes vulpes* or Gray fox *Urocyon cinereoargenteus*) was observed on the Wood Laboratory property at the center of the northern portion of the array. Other common mammal species in the area that may tamper with AudioMoths include Deer mouse (*Peromyscus maniculatus*), Raccoon (*Procyon lotor*), and Virginia opossum (*Didelphis virginiana*).

Points were arranged in an irregular grid pattern such that no two points were closer than 35 m. At each point, a treatment and control AudioMoth were deployed on the same tree. AudioMoth orientation (N/S) was randomized between the control and treatment groups to avoid any directional bias. The control AudioMoth received no treatment, while the treatment AudioMoth received either Liquid Fence, Bonide, or a cayenne pepper and Vaseline mixture. Assignment of each of the three treatments to a point was also randomized. At 34 points, the treatment AudioMoth received the cayenne pepper and Vaseline mixture; at 41, Liquid Fence; and at 40, Bonide. The treatments were applied in the field after AudioMoths were zip-tied to trees approximately 1.5 m off the ground. Bonide and Liquid Fence were sprayed once onto the housing apparatus, and a mixture of cayenne pepper and Vaseline was applied in an approximately 1 × 2 in strip, one inch above and below the AudioMoth. The cayenne pepper and Vaseline mixture was not applied directly to the AudioMoth bag because the consistency of the material may affect sound transmission to the recorder microphones.

The Bonide spray used was the Shot-Gun^®^ Repels-All^®^ Animal Repellent Spray manufactured by Bonide^®^ Products LLC, Oriskany, NY, USA. Its active ingredients consist of putrescent whole egg solids, cloves, and garlic oil. Its inert ingredients consist of fish meal, fish oil, magnesium silicate, magnesium sulfate, meat meal, sodium benzoate, urea, vinegar, water, and wintergreen oil. The solution is reported to be resistant to rain and to last for two months, though for maximum effectiveness it may require reapplication every 7–10 days [16]. The Liquid Fence spray was the Liquid Fence^®^ All-Purpose Animal Repellent Ready-to-Use, manufactured by Spectrum Brands, Earth City, MO, USA. Its ingredients consist of cornmint oil, cinnamon oil, castor oil, garlic oil, water, polyglyceryl oleate, and xanthan gum. The solution is reported to require reapplication after heavy rainfall but otherwise lasts for four weeks [17].

### 2.3. Damage Scoring

AudioMoth damage was assessed in the lab by a single observer on a scale of 0–3 after retrieval. The observer was blind to treatment type during scoring. A score of 0 indicated no mammal damage; a score of 1 indicated 1–2 small bites but no significant damage to the reclosable bag; a score of 2 indicated multiple small holes in the reclosable bag that would have allowed moisture into the housing; and a score of 3 indicated holes larger than 13 × 13 mm that would have allowed significant moisture into the housing. Damage to the chiffon bags was not included in the damage scoring analysis.

### 2.4. Data Analysis

Analysis was conducted in R Version 2026.01.1 [18]. We used a two-sided Wilcoxon signed-rank test to assess a significant difference in damage levels between treatment and control AudioMoths within treatment groups. We used a Bonferroni-corrected alpha of 0.0167 to correct for the three hypothesis tests.

## 3. Results

**Figure 3:**
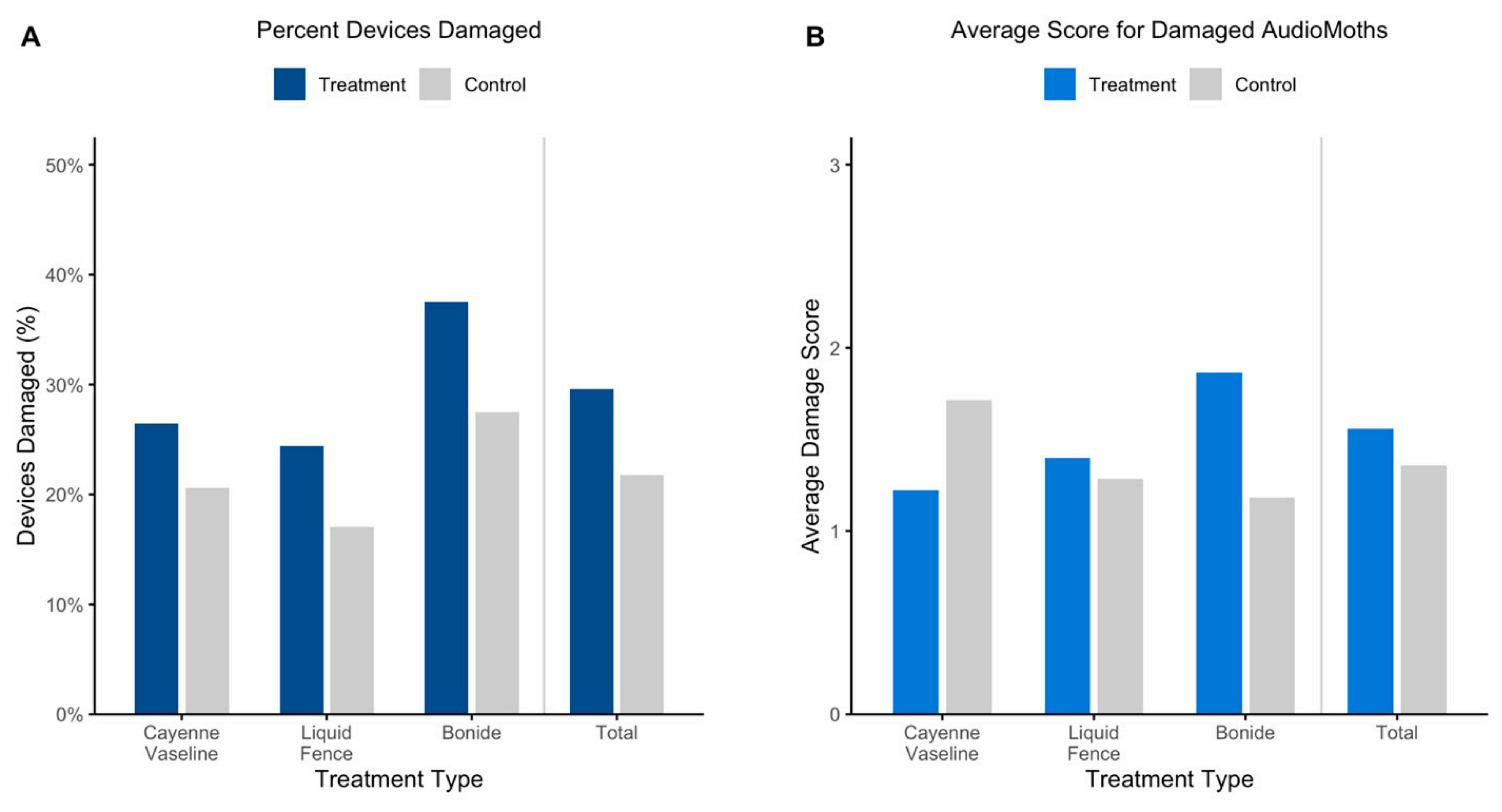
Damage comparisons for treatment vs. control AudioMoths. (**A**) The percentage of devices damaged in the treatment vs. control pairings for each treatment type and total. (**B**) The average damage score for damaged AudioMoths for each treatment type and total. A vertical line divides the per-deterrent treatment and control comparison from a pooled comparison.

In total, 59 of the 230 AudioMoths deployed sustained chewing damage from mammals. Of the 59 damaged, 25 were controls, 15 were treated with Bonide, 10 were treated with Liquid Fence, and 9 were treated with the cayenne Vaseline, corresponding to a damage rate of 21% for control AudioMoths and 29% for treated AudioMoths. Of the 34 damaged treatment AudioMoths, 18 received a score of 1; 13 received a score of 2; and 3 received a score of 3. Of the 25 damaged control moths, 18 were assigned a score of 1; 5 were assigned a score of 2; and 2 were assigned a score of 3. Treated AudioMoths were thus both damaged at a higher rate than control AudioMoths and had higher average scores when damaged (Figure 3).

For all treatment groups, the paired Wilcoxon signed-rank test indicated no significant difference between damage scores in control and treatment pairs at a Bonferroni-corrected alpha of 0.0167 (Table 1). We note that AudioMoths treated with Bonide had higher damage scores than their paired controls resulting in a *p*-value that would have been statistically significant without a Bonferroni correction (*p* = 0.028).

## 4. Discussion

In total, 59 of the 230 AudioMoths deployed sustained chewing damage from mammals, and in 42 of the pairs, there was a non-zero difference in damage between the control and treatment AudioMoths. There was no significant difference in damage between treatment and control AudioMoths within treatments. However, under all three treatments, treated AudioMoths were damaged at a higher rate than control AudioMoths. Our results thus provide no evidence of deterrents decreasing chewing damage, and in fact suggest that such treatments may increase damage to AudioMoth bags. This may be because the treatments we used deterred some mammals but were less effective against the specific species responsible for the bulk of the damage, such as small rodents. It is also possible that the smell from the deterrents initially attracts mammals, but as the time post-application increases, the treatment becomes less effective at deterring chewing after attraction.

This simulated deployment differed from our traditional deployments in multiple ways, all of which may affect absolute damage rates observed, but are unlikely to affect our conclusions regarding the efficacy of deterrents. We only deployed AudioMoths for one month, but true deployments typically last longer. We deployed recorders in the fall, which may have different levels of mammal activity compared to usual summer deployments. We typically reuse the chiffon bags that hold the reclosable bags between deployments, and they may carry a scent which attracts mammals differently based on what the chiffon bag has weathered in its previous deployment. When an AudioMoth is recording, a small LED light on the unit blinks; these AudioMoths were not on, so the attractiveness of this light was not evaluated.

It is possible that our deterrent application methods may have contributed to their ineffectiveness. We opted to apply the cayenne mixture above and below the recorder on the tree because we were concerned that applying it directly to the recorder might block sound, and that collecting and processing the recorders at the end of the deployment would be complicated if they were covered in the mixture. This method may not have been particularly deterring to mammals. Additionally, 13 days of light rain during our deployment may have removed the deterrent from our treated AudioMoth bags. Bonide is reported to last through rain, but the manufacturer of Liquid Fence suggests reapplication after heavy rain. Both manufacturers do suggest reapplying the spray deterrents weekly for maximum effectiveness, but we consider our field trial to reflect a realistic field deployment, in which it would generally not be practical to return to an array to re-treat devices after rain events or between weeks. Future studies may have greater success with reapplication or deterrents with different formulations.

Chewing damage from mammals will become an increasingly costly problem as sensors become cheaper, allowing for larger sensor deployments, which will incentivize users to reduce costs by using inexpensive sensor housing. Commercial mammal deterrents provide a seemingly promising approach to avoiding damage, but we find that chewing behaviors were not abated by the application of three different deterrents and may have, in fact, been increased. Future research should examine other deterrent methods, different methods of application for the deterrents tested here, and different experimental designs.

## Figures and Tables

**Table 1:** Treatments, reported *p*-values, and test statistics for the Wilcoxon signed-rank test.

Treatment	*p*	*V*
Liquid Fence	0.236	46
Bonide	0.028	135
cayenne Vaseline	0.971	44.5

## Data Availability

All data and results are available at https://github.com/brookedg/mammal_deterrents.
